# Hepatic gene expression explains primary drug toxicity in bipolar disorder

**DOI:** 10.1038/s41398-019-0666-4

**Published:** 2019-12-09

**Authors:** Anna Maria Birkl-Toeglhofer, Christoph Birkl, Ida Cirila Llenos, Serge Weis, Johannes Haybaeck

**Affiliations:** 1 Diagnostic & Research Institute of Pathology, Medical University of Graz, Graz, Austria; 2 Department of Pathology, Neuropathology and Molecular Pathology, Medical University of Innsbruck, Innsbruck, Austria; 3 Department of Neurology, Medical University of Graz, Graz, Austria; 4 The Stanley Medical Research Institute, Chevy Chase, MD USA; 5 Division of Neuropathology, Department of Pathology and Neuropathology, Neuromed Campus, Kepler University Hospital, Medical School, Johannes Kepler University, Linz, Austria; 6 Departments of Psychiatry and Pathology, Uniformed Services University of the Health Sciences, Bethesda, MD USA; 7 Center for Biomarker Research in Medicine, Graz, Austria; 8 Department of Pathology, Medical Faculty, Otto von Guericke University Magdeburg, Magdeburg, Germany

**Keywords:** Pathogenesis, Bipolar disorder

## Abstract

In bipolar disorder (BPD), long-term psychotropic drug treatment is often necessary to prevent relapse or recurrence. Nevertheless, adverse drug effects including disturbances in hepatic metabolism are observed and still poorly understood. Here, the association between hepatic gene expression and histopathological changes of the liver was investigated. By the use of microarrays (Affymetrix U133 plus2.0), a genome-wide expression study was performed on BPD patients with psychotropic drug treatment (*n* = 29) compared to unaffected controls (*n* = 20) and validated by quantitative real-time PCR. WebGestalt was used to identify over-represented functional pathways of the Reactome database. Association analyses between histopathological changes and differentially expressed genes comprised in the over-represented functional pathways were performed using regression analyses, from which feature-expression heatmaps were drawn. The majority of identified genes were underexpressed and involved in energy supply, metabolism of lipids and proteins, and the innate immune system. Positive associations were found for genes involved in all pathways and degenerative changes. The strongest negative association was observed between genes involved in energy supply and hepatic activity, as well as inflammation. In summary, we found a possible association between gene expression involved in various biological pathways and histopathological changes of the liver in BPD. Further, we found support for the probable primary toxic effect of psychotropic drugs on hepatic injury in BPD. Even if the safety of psychotropic drugs improves, adverse effects especially on hepatic function should not be underestimated.

## Introduction

Bipolar disorder (BPD) is a severe mental illness characterized by recurrent and chronic fluctuations of manic and depressive episodes. More than 1% of the population is affected by BPD regardless of nationality, ethnicity or socioeconomic status^1^. To prevent relapse or recurrence in patients suffering from BPD, long-term drug treatment is often necessary^2,3^. Psychotropic drugs encompass antipsychotics, antidepressants and mood stabilizers including anticonvulsant drugs and lithium. Although the treatment aims to improve or resolve mood symptoms and prevent future relapses, adverse effects during the long-term drug treatment in BPD have been observed and reviewed^4,5^. Adverse effects, like renal, thyroid and parathyroid malfunction^6^, weight gain^7^, and hepatoxicity^8^ were described. An induction of hepatoxicity by elevated liver enzymes due to psychotropic drug treatment was reported for antidepressants, antipsychotics, mood stabilizers, and anxiolytic agents^9^.

In an animal study, adverse effects after psychotropic drug treatment on the liver were shown^10^. Sprague-Dawley rats receiving concomitantly valproic acid and a high fat diet revealed an impaired hepatocellular mitochondrial function, an increased free fatty acid accumulation and a more severe hepatotoxicity in comparison to fat diet only. Even if the safety of psychotropic drugs has improved, hepatic injury is not precluded and adverse effects of psychotropic drugs should not be underestimated.

As the liver is the main site for metabolism including drug metabolism, disturbances in hepatic metabolism might result in non-alcoholic fatty liver disease (NAFLD). NAFLD ranges from simple steatosis to non-alcoholic steatohepatitis (NASH) in individuals with absent or non-excessive alcohol consumption^11,12^. Hepatic steatosis is characterized by the accumulation of lipids in the cytoplasm of hepatocytes^13,14^. Here, particularly an imbalance of triglyceride synthesis and triglyceride breakdown is present. The progressed form, NASH, shows hepatocellular injury, referred to as hepatocellular ballooning, and is accompanied by inflammatory infiltrates, as well as by the accumulation of extracellular matrix proteins^15,16^. Progressed forms with inflammation and varying stages of fibrosis might further progress to liver cirrhosis and eventually to hepatocellular carcinoma (HCC).

Data from several studies indicate an association between an increased prevalence of liver disease and mood disorders^17–20^. A study assessing the burden of medical comorbidities in BPD showed an 3.97 higher odds ratio for liver disease compared to controls^17^. Similarly, a study of veterans with BPD reported a 21.5% prevalence and a 2.27 higher odds ratio for any liver disease compared to matched controls without BPD^18^. In addition, increased lifetime rates of depression and anxiety disorders were found in subjects suffering from NASH^19^. Another study showed an association between severity of depressive symptoms and hepatocellular ballooning in NAFLD^20^. Patients suffering from subclinical or clinical depression revealed a 2.1 times and 3.6 times higher likelihood of more severe hepatocyte ballooning, respectively.

Several molecular pathways were found to be involved in NAFLD^21,22^. A gene expression study distinguishing between low and high liver fat content of human liver biopsy tissue revealed an involvement of pathways related to carbohydrate, lipid and amino acid metabolism, insulin signaling, inflammation, and MAPK signaling^21^. A meta-analysis of several publicly available gene expression datasets investigating NAFLD progression revealed a signature set of 218 genes associated with histologically assessed features^22^. To which extent psychotropic drugs in BPD influence hepatic gene expression signatures and, hence, interfere or impair normal hepatic function, is still poorly understood.

Therefore, this study examines hepatic gene expression in individuals with BPD treated with psychotropic drugs compared to controls. The association between histopathological change and differentially expressed genes of the liver was investigated.

## Materials and methods

### Post-mortem human liver samples

In the present study, post-mortem liver samples were examined from 29 individuals with BPD and 20 controls. The liver samples were obtained from the Stanley Medical Research Institute (Chevy Chase, MD, USA). Details on demographics, clinical data, and storage information were reported elsewhere^23^. A public statement about the Stanley Brain Collection from January 2007 (updated August 2009) is available online (www.stanleyresearch.org). Permission for donation and further information about the deceased was obtained from the next of kin. Tissue samples were collected by trained medical examiners according to a standardized procedure. The clinical diagnosis was obtained from the medical examiners report; a structured interview was conducted with next of kin some time after the death of the patient. The diagnostic criteria of DSM IV were applied. The summarized subject characteristics are shown in Table 1. The study was approved by the local ethics committee of the Medical University of Graz (31–41 ex 18/19).

**Table 1:** Characteristics of subjects with bipolar disorder and the control group.

	Bipolar disorder(*n* = 29)	Control group(*n* = 20)	*P*
Age, years, mean (±SD)	41.8 (±14.93)	45.9 (±11.13)	0.276
Gender, n (%)			
Female	15 (51.7)	4 (20.0)	0.037
Male	14 (48.3)	16 (80.0)	
BMI, kg/m², mean (±SD)	30.8 (±6.82)	27.8 (±6.34)	0.123
PMI, hours, mean (±SD)	39.1 (±20.36)	23.1 (±10.48)	0.002
Medication, *n* (%)			
Antidepressants	15 (51.7)		
Antipsychotics	16 (55.2)		
Mood stabilizers	21 (72.4)		
Anticholinergic	1 (3.5)		

*BMI* body mass index, *PMI* post-mortem interval

### Histological assessment of liver samples

Formalin-fixed and paraffin-embedded sections of 5 µm were stained for 25 BPD subjects and 17 controls. The histopathological parameters were divided into six main categories: (1) degenerative changes, (2) hepatic reactivity, (3) inflammation, (4) fibrosis, (5) endogenous reaction, and (6) steatosis. The corresponding grading scheme is shown in Supplementary Table 1. The histological analysis for various histological parameters was performed by two experienced pathologists blinded to the diagnosis (ICL, JH).

### Microarray analysis

The sample preparation and microarray processing were performed at the Microarray Core Facility of the Johns Hopkins University (Baltimore, MD, USA). Briefly, total RNA was isolated from frozen liver tissue using TRIzol (Invtitrogen, Carlsbad, CA, USA) and chloroform, followed by the precipitation with isopropyl alcohol, washing with 70% ethanol and dissolving in DEPC water. Additionally, RNA was purified using RNeasy column purification (Qiagen, Valencia, CA, USA). The quality of total RNA was assessed on an Agilent Bioanalyzer (Agilent Technologies, Palo Alto, CA, USA) using the RNA integrity number (RIN) with a mean of 5.3 and a standard deviation of 2.2 of all samples. The gene expression analysis was performed using the Human Genome U133 Plus 2.0 array (Affymetrix, Santa Clara, CA, USA).

### Data normalization and differential gene expression analysis

All CEL files were normalized using the Robust Multi-Array Average (RMA) algorithm to control for array-to-array variation. After normalization, non-specific filtering was performed including the removal of probes with a low overall intensity (<log_2_(100) in a minimum of 10% of the samples) and a low variability (log_2_ intensities interquartile range of <0.5). Principal component analysis (PCA) was performed to test the overall variability of the samples. A linear model and moderate *t*-statistic was applied to assess differences in gene expression between BPD and control sample. To adjust for confounding effects the model included the following demographic and clinical factors: age, gender, body mass index (BMI) and post-mortem interval (PMI) in hours. The Benjamini and Hochberg adjustment was applied to control for multiple testing. Differentially expressed genes were considered statistically significant with a fold change of 1.5 and a false discovery rate (FDR) of <5%. A heatmap of the differentially expressed genes was generated. Data normalization and differential gene expression analysis were conducted in R 3.4.1 (R Foundation for Statistical Computing, Vienna, Austria) using the packages affy^24^, genefilter^25^, biomaRt^26^ and limma^27^. The dataset is available online at www.stanleyresearch.org. The code is available upon request.

### Enrichment analysis

Differentially expressed genes were analyzed using the WEB-based Gene SeT AnaLysis Toolkit (WebGestalt)^28^. To identify over-represented functional pathways in liver tissue of BPD subjects, an enrichment analysis was performed using the functional database Reactome. The analysis parameters included categories with five to 1000 involved genes and a FDR adjusted *P* = <0.05 for the Benjamini and Hochberg correction.

### Visualization of feature-expression heatmaps

Feature-expression heatmaps were generated according to a previously described approach^29^. Only BPD samples were included for the visualization. Histologically assessed features were grouped into degenerative changes, hepatic activity, endogenous hepatic activity, steatosis, fibrosis, and inflammation. Depending on the data type of the histological parameter, ordinal logistic regression or binary logistic regression was performed for the association analyses between histological parameters and log_2_ intensity values of the top 10 differentially expressed genes obtained from the over-represented functional pathways analysis. The effect size, standard error, *p*-value and the FDR adjusted *p*-value from the Benjamini and Hochberg correction were calculated and plotted as feature-expression heatmaps using R. An FDR adjusted *P* < 0.2 was considered statistically significant. Heatmap, significance, and FDR plots were merged using MATLAB 2012b (The MathWorks, Inc., Natick, Massachusetts, USA).

### Validation by quantitative real-time PCR

Total RNA was extracted from frozen post-mortem liver tissue from a subset of the BPD (*n* = 16) and control group (*n* = 14). The RNA isolation was performed using TRIzol Reagent (Thermo Fisher Scientific, Waltham, MA, USA) and a subsequent high-salt precipitation using 1.2 M sodium chloride and 0.8 M sodium citrate. The RNA quantity and quality was assessed with the NanoDrop (Thermo Fisher Scientific, Waltham, MA, USA). Reverse transcription was performed using the High Capacity cDNA Reverse Transcription Kit (Applied Biosystems, Foster City, CA, USA). For quantitative real-time PCR (qRT-PCR), 10 ng cDNA was mixed with the Luna Universal qPCR Master Mix (New England Biolabs, Ipswich, MA, USA) and gene specific primers according to the manufactures protocol. The gene expression was assessed in 384-well plate format with the QuantStudio real-time PCR system (Applied Biosystems, Foster City, CA, USA). The cycle condition were 2 min at 50 °C, 10 min at 95 °C followed by 45 cycles with 15 s at 95 °C and 1 min at 60 °C. The target genes for validation were chosen based on identified differential expression in the microarray analysis. The corresponding primers are listed in Supplementary Table 2. The amplicon size was validated by an 2% agarose gel stained with HDGreen Plus DNA Stain (Intas Science Imaging, Goettingen, Germany). In total, 10 µl of amplification product was loaded into each well, the separation was performed for 30 min at 100 V and the bands were detected by the Gel iX 20 Imager (Intas Science Imaging, Goettingen, Germany). Relative gene expression was assessed by using the 2^−ΔΔCt^ method^30^. Triplicates of each sample were used to calculate the average Ct value. For normalization, the mean of the two endogenous control genes, *RPL41* and *IPO8*, was used. Only samples with a specific amplicon indicated by an appropriate dissociation curve were included for further analysis.

### Statistical analysis

Differences of demographic and clinical characteristics between the groups were tested for significance using the unpaired T test for normally distributed data. The Fisher’s exact test was used for binary data. The histopathological parameters were tested using the non-parametric Mann–Whitney U test. Based on the data distribution, differences of the genes fold change values assessed by qRT-PCR between the groups were tested using the unpaired T test or Mann–Whitney U test. Pearson correlation analysis between microarray and qRT-PCR data was performed. Statistical analysis was performed using R 3.4.1 (R Foundation for Statistical Computing, Vienna, Austria) and GraphPad Prism 5 (GraphPad Software, San Diego, CA, USA). The significance threshold was set at *P* = 0.05.

## Results

### Subject characteristics

Liver tissue of deceased subjects with the clinical diagnosis of BPD and controls with no known history of psychiatric disorder was included in the study. Neither the BPD subjects, nor the controls were suffering from viral hepatitis. One BPD subject was diagnosed with diabetes mellitus type 1. The control sample included predominantly male subjects. No significant difference in age or BMI between BPD subjects and controls was observed. The mean PMI was significantly higher in BPD than in the control group.

### Histopathologic changes

The frequency distributions of all assessed histopathological parameters are shown in Supplementary Table 1. All individuals in the BPD and the control group showed steatosis except for one control. Steatohepatitis was present in 58.6% of the BPD group and 35.2% of the controls (*P* = 0.405). In the category of degenerative changes, controls showed a higher degree of parenchymal iron pigment storage (*P* < 0.001) compared to BPD subjects. In the category of inflammation, a higher grade of portal granulocytes (*P* = 0.025) and lobular histiocytes (*P* = 0.010) was observed for BPD subjects, whereas the grade of lobular CD8^+^ cells (*P* = 0.001) was higher in controls. None of the remaining parameters across all categories showed a significant difference between BPD subjects and controls.

### Gene expression

**Fig. 1:**
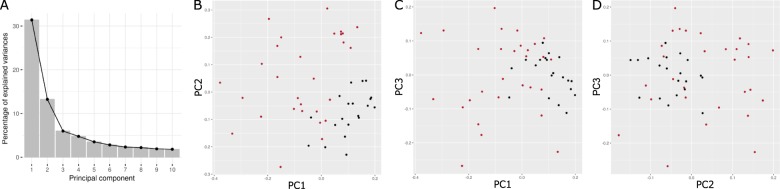
Principle components analysis of gene expression profiles in bipolar disorder and controls. **a** Scree plot with the first 10 principle components (PC). PC 1, 2 and 3 explain 32%, 13 and 6% of the variance in the samples, respectively. **b**–**d** Every point represents a transcriptome of BPD subjects (red) and controls (black). PC1 against PC2 is plotted in (**b**), PC1 against PC3 in (**c**), and PC2 against PC3 in (**d**).

**Fig. 2:**
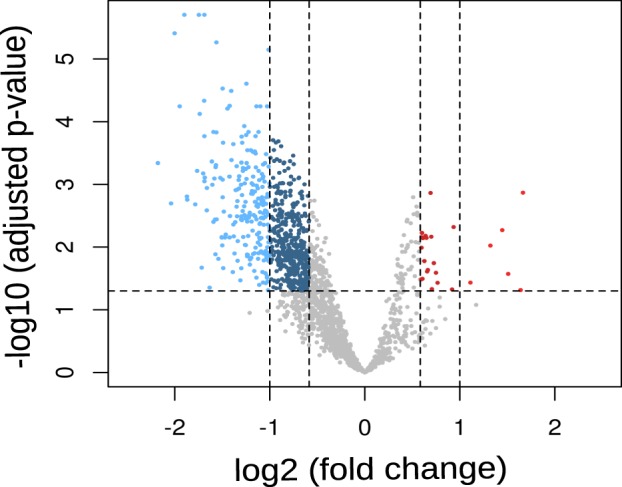
Volcano plot displaying differential expressed genes between BPD and control sample. Each dot represents a probe set plotted according to the log_2_ fold change on the *x*-axis and the negative logarithm of the *P* on the *y*-axis. Downregulated genes are shown in blue (FC −1.5 and FDR < 0.05) while up-regulated genes (FC 1.5 and FDR < 0.05) are shown in red. BPD bipolar disorder, FC fold change, FDR false discovery rate.

The overall gene expression variability of the samples is visualized in the scree plot and the PCA plots in Fig. 1. Principal component one to three explained 31.7%, 13.0%, and 6.3%; respectively, amongst all samples. A clear difference in gene expression is shown in the first principal component between BPD and controls. A total of 7280 gene probes remained after non-specific filtering of the microarray data. Of these, 648 differentially expressed genes met the statistical significance threshold and the fold change criteria. Differentially expressed genes are displayed in the volcano plot, see Fig. 2. Substantially more genes were found to be underexpressed (*n* = 623) than overexpressed (*n* = 25) in BPD compared to controls. A table of all significantly differentially expressed genes is provided in Supplementary Table 3 listing the gene annotation, FC and FDR.

### Pathway analysis

All differentially expressed genes meeting the significance criteria were used to identify molecular pathways most probably affecting the hepatic gene expression in BPD. The over-represented genes analyzed using WebGestalt and the Reactome database revealed nine significant functional pathways related to metabolism and the innate immune system, shown in Table 2. Of these pathways, four are related to the respiratory electron transport chain (RETC), two are related to the lipid metabolism, two are related to the amino acid metabolism including the ornithine decarboxylation regulation, and one is related to neutrophil degranulation.

**Table 2:** Over-represented functional pathways from differentially expressed genes in bipolar disorder.

Reactome pathway	N of genes in pathway	N of DEGs involved	FDR
Respiratory electron transport, ATP synthesis by chemiosmotic coupling, and heat production by uncoupling proteins (R-HSA-163200)	112	21	1.63 × 10^−5^
Respiratory electron transport (R-HSA-611105)	91	17	2.22 × 10^−4^
The citric acid cycle and respiratory electron transport (R-HSA-1428517)	155	22	4.16 × 10^−4^
Neutrophil degranulation (R-HSA-6798695)	447	42	5.82 × 10^−4^
Metabolism of lipids and lipoproteins (R-HSA-556833)	739	59	7.72 × 10^−4^
Complex I biogenesis (R-HSA-6799198)	51	10	1.41 × 10^−2^
Biological oxidations (R-HSA-211859)	175	20	1.48 × 10^−2^
Metabolism of amino acids and derivatives (R-HSA-71291)	316	29	2.01 × 10^−2^
Regulation of ornithine decarboxylase (R-HSA-350562)	47	9	2.69 × 10^−2^

*DEGs* differentially expressed genes, *FDR* false discovery rate

### Association between histological changes and expression of RETC related genes

**Fig. 3:**
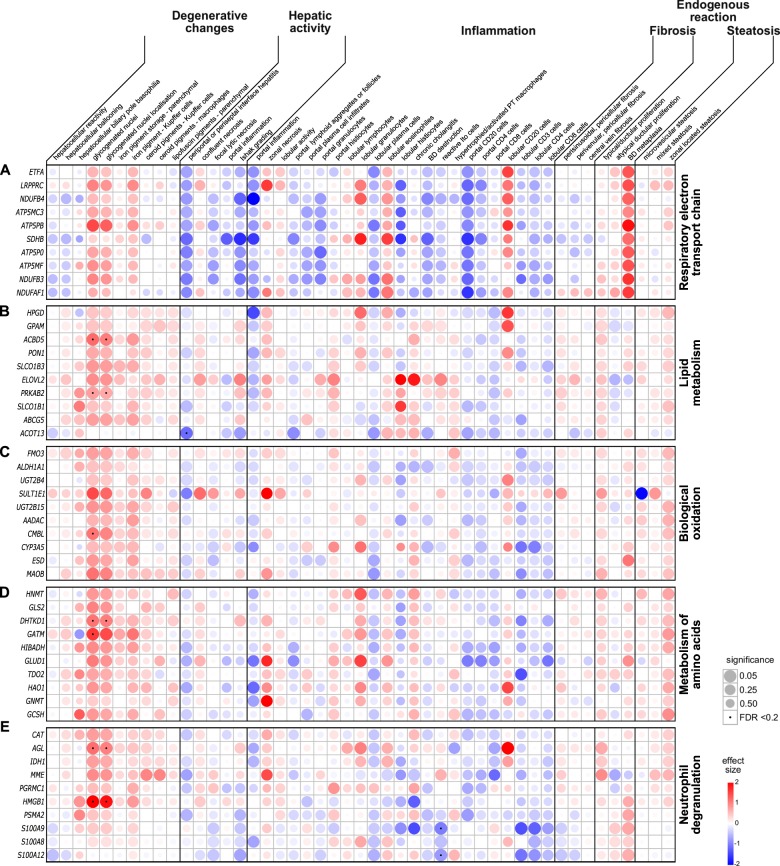
Feature-expression heatmaps of hepatic histopathological features and expression of genes related to metabolic and innate immunity system pathways (**a**–**d**). The histopathological features were on ordinal or binominal scale. The gene expression was expressed as log_2_ intensity values. Statistical analyses were performed using ordinal or binary logistic regression. The intensity of the color represents the regression coefficients (red = positive, blue = negative). The circle radius displays the statistical significance. Dotted circles represent a false discovery rate (FDR) below 0.2 for multiple testing. See Supplementary Table 3 for gene abbreviations.

The associations between histologic changes of the liver and the top 10 differentially expressed genes of the RETC pathway are shown in Fig. 3a. None of the analyzed associations were significant after FDR correction. Despite the lack of associations clustering for degenerative changes, glycogenated nuclei, and their localization revealed a strong positive association. Features of hepatic activity displayed a cluster of negative associations with RETC related genes, out of all the Ishak grading revealed the strongest negative association. In the group of endogenous hepatic reaction, the bile duct metaplasia showed a positive association with RETC related genes. In the steatosis feature group, a positive association was observed between zonal distributed steatosis and RETC related genes. A weak negative association clustering was found for features of fibrosis. Several features of inflammation showed a negative association, namely portal inflammation, portal lymphoid aggregates, plasma cell infiltrates, granulocytes, CD20 positive B-cells and CD4 positive T-cells, lobular granulocytes and histiocytes, chronic cholangitis, bile duct destruction, reactive Ito cells, CD3, CD4 and CD8 positive T-cells. Positive associations were found for lobular plasma cells, eosinophils and CD20 positive B-cells and genes belonging to the RETC pathway.

### Association between histological changes and expression of lipid metabolism related genes

The feature-expression heatmap depicting the associations between histological parameters and expression of genes related to lipid metabolism is displayed in Fig. 3b. The heatmap revealed a cluster of positive associations between degenerative changes and gene expression related to lipid metabolism. The genes coding for the acyl-CoA binding domain containing 5 (*ACBD5*) and the protein kinase AMP-activated non-catalytic subunit beta 2 (*PRKAB2*) associated significantly positively with glycogenated nuclei and their localization. Although a clustering of associations for hepatic activity and lipid metabolism was absent, the acyl-CoA thioesterase 13 (*ACOT13*) expression and periportal or periseptal interface hepatitis revealed a significant negative association. Clustering of associations for the histological features of endogenous hepatic reaction, steatosis, fibrosis, and inflammation could not be recognized. None of these analyses were significant after FDR correction.

### Association between histological changes and expression of biological oxidation related genes

The feature-expression heatmap in Fig. 3c displays the associations between histological parameters and expression of genes involved in biological oxidations. Genes of the biological oxidations pathway are involved in functionalization and conjugation of various chemical compounds. Sub-pathways include the biological oxidation of xenobiotics and endogenous compounds via the cytochrome P450 system, amine oxidase reaction, glucuronidation, and others. The features of degenerative changes and genes of biological oxidations revealed a cluster of positive associations. The gene coding for the carboxymethylenebutenolidase homolog (*CMBL*) associated significantly positively with glycogenated nuclei. None of the other analyses revealed significance after FDR adjustment. Association clustering was absent in other histological changes groups.

### Association between histological changes and expression of amino acid metabolism related genes

The associations between histological changes and genes involved in the metabolism of amino acids are shown in Fig. 3d. A cluster of positive associations between degenerative changes and gene expression related to amino acid metabolism was observable. Statistically significant positive associations were observed for glycogenated nuclei and their localization and the genes dehydrogenase E1 and transketolase domain containing 1 (*DHTKD1*) and glycine amidinotransferase (*GATM*). None of the other analyses revealed significance after FDR correction. Clustering of associations for the histological changes of hepatic activity, endogenous hepatic reaction, fibrosis and inflammation could not be recognized. Noteworthy, features of steatosis revealed a positive association clustering, even though not statistically significant.

### Association between histological changes and expression of neutrophil degranulation related genes

The feature-expression heatmap in Fig. 3e depicts a cluster of weak positive associations between histological changes and expression of genes related to neutrophil degranulation. The genes amylo-alpha-1, 6-glucosidase, 4-alpha-glucanotransferase (*AGL*) and high mobility group box 1 (*HMGB1*) associated significantly positively with glycogenated nuclei and their localization. Associations clustering for the histological features of hepatic activity, endogenous hepatic reaction, fibrosis, and inflammation could not be recognized. A positive significant association was observed for reactive Ito cells and the S100 calcium binding protein A12 (*S100A12*) gene. Any of the other analyses reached statistical significance after FDR adjustment.

### Validation of differentially expressed genes

To verify differential expression identified by microarray analysis, qRT-PCR was performed for representative genes from the functional pathways. The amplicon size verification is shown in Supplementary Fig. 1. Among the 14 selected genes, statistically significant altered mRNA levels in BPD were confirmed for 11 genes. Significantly decreased mRNA levels were found for ATP synthase peripheral stalk-membrane subunit b (*ATP5PB*), succinate dehydrogenase complex iron sulfur subunit B (*SDHB*), *ACOT13*, arylacetamide deacetylase (*AADAC*), carboxymethylenebutenolidase homolog (*CMBL*), 3-hydroxyisobutyrate dehydrogenase (*HIBADH*), tryptophan 2,3-dioxygenase (*TDO2*), hydroxyacid oxidase 1 (*HAO1*) and catalase (*CAT*). mRNA levels of *S100A9* and *S100A12* were significantly increased. All qRT-PCR results are shown in Supplementary Fig. 2.

## Discussion

In this study, a gene expression approach was used to investigate effects of psychotropic drugs on liver tissue in BPD subjects compared to controls. To the best of our knowledge, this is the first gene expression study investigating psychotropic drug treatment in post-mortem liver tissue of BPD patients. Biological pathways related to energy supply by the RETC, lipid and amino acid metabolism, biological oxidation and neutrophil degranulation were found to be over-represented among the mainly underexpressed DEGs. The association analysis between hepatic histopathological changes and expression of genes included in the addressed biological pathways can be regarded as a next step to elucidate side effects of psychotropic drug treatment in BPD.

Both groups, BPD and controls, revealed liver injury assessed by diverse histopathological parameters including fat accumulation and inflammation. In addition, the BMI was comparably high for both groups. A previous study showed a five to nine-fold increased risk for NAFLD and NASH with a BMI above 30 kg/m² ^31^. This finding is in agreement with the results found in our study. Although the groups showed histopathological similarities, differences in gene expression could be observed. This supports our hypothesis of a substantial influence of psychotropic drug treatment on hepatic gene expression in BPD. We anticipate that by including healthy liver tissue as control, an even stronger effect would be exposed on gene expression level.

A decreased hepatic expression of biological oxidations related genes and especially a decrease of RETC related genes were observed in BPD. Mitochondrial dysfunction has been extensively reviewed and plays a central role in NAFLD^32^. Nevertheless, a more intense effect was observed in liver tissue of BPD when compared to the controls with resembling liver injury. Examining brain tissue of BPD individuals revealed similar findings regarding an impaired mitochondrial function^33^. Another study investigating brain tissue of BPD and schizophrenia individuals observed altered RETC gene expression which was supposed to be due to antipsychotic drug treatment^34^. In contrast, a previous gene expression study conducted with whole blood samples of BPD individuals with current depressive state showed an increased expression of genes related to the electron transport chain^35^. Due to the unavailability of the current psychiatric state (mania, euthymia, depression) of each individual before death, an exclusion or specific investigation of potential state-related effects is not possible in this study. We suppose that the reduced expression of RETC related genes resembles a diminished supply with energy for hepatic cells upon psychotropic drug treatment and, hence, a more drastic effect on liver injury in BPD.

As a disturbed lipid metabolism is one of the major hallmarks in NAFLD^12–14^, our data of downregulated lipid metabolism genes are in line with the literature. The association between glycogenated nuclei, which are often found in NAFLD^11^, and *ACBD5* upon psychotropic drug treatment was not reported yet, to the best of our knowledge. The ACBD5 protein is involved in peroxisomal tethering to the endoplasmatic reticulum together with vesicle-associated membrane-protein-associated proteins^36^. This binding function was shown to be involved in lipid homeostasis and peroxisomal maintenance through the exchange of lipids between peroxisomes and the endoplasmatic reticulum. A recent study showed that *ACDB5*-deficiency in a patient led to an accumulation of very long-chain fatty acid in plasma resulting from an impaired β-oxidation^37^. In a recent study, epoxide hydrolase 2 (*EPHX2*) expression was decreased in liver tissue of male rabbits treated with the antidepressant drug sertraline, where a reduced metabolizing capacity of drugs and fatty acids was hypothesized as potential basis of hepatoxicity^38^. This observation is in accordance with our finding on decreased *EPHX2* expression in human liver. In addition, *PRKAB2* expression was found to be associated with glycogenated nuclei. Interestingly, a previous study found an association between weight gain upon clozapine or olanzapine treatment in patients with schizophrenia and schizoaffective disorder and a *PRKAB2* mutation^39^. Overall, the effect of psychotropic drugs on lipid metabolism seems to be complex and might be underestimated. Our data and the aforementioned studies raised the importance to consider also genetic predisposition in future studies.

Genes related to the amino acid metabolism were underexpressed in BPD. Here, the decreased expression of genes *DHTKD1* and *GATM* were associated with glycogenated nuclei. *DHTKD1*, involved in lysine metabolism, was shown to reduce mitochondrial activity upon inhibition and might influence the development of diabetes^40^. Likewise, reduced *GATM* expression levels were indicated to affect the development of diabetes in a murine diabetes model^41^. This leads to the assumption that BPD individuals with psychotropic drug treatment seem to be more prone to develop diabetes compared to controls with resembling liver injury.

In this study we could show an underexpression of neutrophil degranulation related genes. A diminished neutrophil degranulation does not exclude a potential neutrophil accumulation in the liver. Beside a decreased degranulation potential of neutrophils, an increased expression of S100 calcium binding protein family members was observed. It has been shown that the proteins S100 calcium binding protein A8 (S100A8) and S100 calcium binding protein A9 (S100A9) are strong stimulators of neutrophils and are linked to neutrophil chemotaxis^42^. In this context an association with various malignancies has been reviewed^43,44^ and increased S100A8 and S100A9 serum levels were found in patients with NASH^45^. In addition, enhanced S100A8 and S100A9 expression on protein and mRNA level were reported in HCC and are assumed to promote malignancy progression^46^. This leads to the suggestion that BPD individuals might develop more advanced forms of liver injury. Another study observed elevated levels of S100A8 and S100A9 in plasma of diabetic individuals relating to a glucose sensitive response^47^. With regard to psychotropic drugs, a study on carbamazepine-induced liver injury in mice revealed a time-dependent increase of *S100A8* and *S100A9* mRNA expression levels probably resulting from an elevated production of reactive oxygen species^48^. Our findings might be rather linked to a primary toxic effect than an immunological related effect due to the impaired neutrophil degranulation. This would suggest that an anti-immunological therapy for psychotropic drug side effects in BPD with liver injury might have a more beneficial effect.

In a previous study, an association between psychotropic drug treatment outcome and metabolic disorder in BPD patients was described^49^. This study demonstrates that an increased BMI negatively affects response and remission among patients with lithium and valproate acid treatment. These results also emphasize our finding and therefore the importance to improve negative psychotropic drug side effects affecting in general appropriate liver function in BPD.

As in other gene expression studies, the heterogeneity of human samples and the conglomeration of different hepatic cell types might confine a conclusive result. Apart from the novel insights in hepatic gene expression in BPD using post-mortem tissue, this study had to face the problem that the PMI and, hence, RNA quality, may influence results due to cell autolysis and tissue degradation. Beside the adjustment of probable confounding effects, such as age, gender, and BMI, we also included PMI in the analysis. The RIN values of the samples, although low, are comparable to previous investigation studying post-mortem tissue^50,51^. It has been shown that with increasing PMI, the RIN values are decreasing^51^. Residual factors affecting hepatic gene expression cannot be excluded. These factors might include genetic predispositions and unrecorded clinical factors. In this study, the sample size and the mixed medication history did not allow the categorization based on the psychotropic drug type. For this reason, it became impossible to state direct effects of each medication on hepatic gene expression. Due to the lack of information, state-dependent differences in BPD individuals were not considered in the analysis. With respect to the stated limitations, this study should rather be considered as an exploratory study. Future replication studies, as well as in vitro studies using cell culture and animal models will be needed to deepen the knowledge of hepatic function/dysfunction during psychotropic drug treatment in BPD.

In summary, we showed that psychotropic drug treatment affects different genes and biological pathways in the liver of BPD patients compared to controls. As psychotropic drugs play an essential role in BPD treatment, a better understanding of their effect on hepatic function might reduce the possible risk of liver injury and will help to improve future treatment.

## Supplementary information

Supplementary Figure Legends

Supplementary Figure 1

Supplementary Figure 2

Supplementary Table 1

Supplementary Table 2

Supplementary Table 3
