# Correction: Hepatic gene expression explains primary drug toxicity in bipolar disorder

**DOI:** 10.1038/s41398-020-0749-2

**Published:** 2020-02-17

**Authors:** Anna Maria Birkl-Toeglhofer, Christoph Birkl, Ida Cirila Llenos, Serge Weis, Johannes Haybaeck

**Affiliations:** 1 Diagnostic & Research Institute of Pathology, Medical University of Graz, Graz, Austria; 2 Department of Pathology, Neuropathology and Molecular Pathology, Medical University of Innsbruck, Innsbruck, Austria; 3 Department of Neurology, Medical University of Graz, Graz, Austria; 4 The Stanley Medical Research Institute, Chevy Chase, MD USA; 5 Division of Neuropathology, Department of Pathology and Neuropathology, Neuromed Campus, Kepler University Hospital, Medical School, Johannes Kepler University, Linz, Austria; 6 Departments of Psychiatry and Pathology, Uniformed Services University of the Health Sciences, Bethesda, MD USA; 7 Center for Biomarker Research in Medicine, Graz, Austria; 8 Department of Pathology, Medical Faculty, Otto von Guericke University Magdeburg, Magdeburg, Germany

**Keywords:** Pathogenesis, Bipolar disorder

**Correction to: Translational Psychiatry**

10.1038/s41398-019-0666-4 published online 9 December 2019

The original Article had an incomplete acknowledgements list. The following sentence has now been added to the HTML and PDF versions of this Article:

“This study was performed within the framework of the PhD program Molecular Medicine of the Medical University of Graz. This work was supported by the Medical University of Graz within the Open Access Publishing Funding Program.”

