# Supplementary material for: Hepatic gene expression explains primary drug toxicity in bipolar disorder
Source: Transl Psychiatry. 2019 Dec 9;9:331. doi: 10.1038/s41398-019-0666-4 (PMC6901567; doi:10.1038/s41398-019-0666-4)
Supplement: Supplementary file 1 — Supplementary Figure Legends [file 41398_2019_666_MOESM1_ESM.docx]

**Supplementary Figure Legends**

**Supplementary Figure 1.** Confirmation of amplicon size and primer specificity. The expected amplicon size for each primer pair is shown on a 2% agarose gel. Target genes are indicated above the image and the amplicon size is given in brackets. A list of full gene names is provided in Supplementary Table 3.

**Supplementary Figure 2**. Validation of differentially expressed genes by qRT-PCR in post-mortem liver tissue of BPD and control subjects. Relative gene expression is assessed by using the 2^-ΔΔCt^ method. Expression values were determined by using triplicates and normalized by using the two endogenous control genes *RPL41* and *IPO8*. Based on the data distribution, statistical analyses are performed by using unpaired t-test or Mann Whitney U test. (**A**) Genes of the respiratory electron transport chain: *LRPPRC* expression is decreased in BPD at a trend level (*P*=.073), but was not statistically significant. *ATP5PB* (*P*=.032) and *SDHB* (*P*=.007) mRNA levels are significantly decreased in BPD. (**B**) Genes of the lipid metabolism: *ACOT13* (*P*=.013) expression is significantly decreased in BPD, *PON1* expression is decreased in BPD at a trend level (*P*=.097), but was not statistically significant. (**C**) Genes of biological oxidation: *AADAC* (*P*=.032) and *CMBL* (*P*=.013) expression are significantly decreased in BPD. (**D**) Genes of amino acid metabolism: *HIBADH* (P=.032), *TDO2* (*P*=.002) and *HAO1* (*P*=.037) mRNA levels are significantly decreased in BPD. (**E**) Genes of neutrophil degranulation: *CAT* (*P*=.004) expression is significantly decreased in BPD. A difference in *PSMA2* expression is absent and not statistically significant (*P*=.182). *S100A9* (*P*=.003) and *S100A12* (*P*=.003) mRNA levels are significantly increased in bipolar disorder. See Supplementary Table 2 for gene abbreviations. **P*<0.05, ***P*<0.01.
